# Application of endoscopic third ventriculostomy for treating hydrocephalus-correlated Chiari type I malformation in a single Chinese neurosurgery centre

**DOI:** 10.1007/s10143-017-0844-x

**Published:** 2017-03-22

**Authors:** Yiping Wu, Chuzhong Li, Xuyi Zong, Xinsheng Wang, Songbai Gui, Caiping Gu, Yazhuo Zhang

**Affiliations:** 1 Beijing Neurosurgical Institute, Capital Medical University, Beijing, China; 2 Department of Neurosurgery, Beijing Tiantan Hospital, Capital Medical University, Beijing, China; 3 Beijing Institute for Brain Disorders Brain Tumor Center|, Beijing, China; 4 China National Clinical Research Center for Neurological Diseases, Beijing, China; 5 Department of Neurosurgery, Affiliated Wuxi People’s Hospital of Nanjing Medical University, Wuxi, China

**Keywords:** Endoscopic third ventriculostomy, Hydrocephalus, Chiari type I malformation, Tonsillar herniation

## Abstract

The correlation between hydrocephalus and Chiari type I malformation (CIM) has been debated since Chiari’s first descriptions of CIM but some studies have shown that CIM and hydrocephalus (HCP) could cause symptoms/disease of each other or vice versa. Recent research has found that treatment focused on hydrocephalus with ventricle enlargement also provides alleviation of CIM and even of syringomyelia. However, the lack of consensus among previous studies left unanswered the question of how endoscopic third ventriculostomy (ETV) addresses CIM and why it fails. Ten symptomatic hydrocephalic patients associated with CIM underwent ETV from October 2002 to May 2012. The clinical features and neuroimaging of all patients were reviewed. Statistical analysis was applied to evaluate the changes in the tonsillar ectopia and the ventricle dilation after operation. The mean follow-up period of this series was 92 months (range 24–163 months). Eight patients (80%) remained shunt free or experienced symptom relief following ETV. The remaining two patients were identified as failures due to the deterioration of symptoms or subsequent hindbrain decompression. Endoscopic third ventriculostomy provides an effective treatment for hydrocephalus associated with CIM, which can relieve HCP and improve the symptoms of CIM in most patients. The clinical outcomes are related to the major cause of the tonsillar herniation.

## Introduction

The correlation between hydrocephalus and Chiari type I malformation (CIM) has been debated since Chiari’s first descriptions of CIM. Chiari postulated that the caudal descent of the cerebellar tonsils, which was later named “Chiari type I malformation”, was the result of the supratentorial pressure exerted by long-standing hydrocephalus. However, the large majority of CIM cases presented a normal-sized ventricle [1, 2], so Chiari’s postulation could not reasonably explain the mechanism of the herniation of the cerebellar tonsils. Recent research has found that treatment focused on hydrocephalus with ventricle enlargement also provides alleviation of CIM and even of syringomyelia; thus, there is a consensus among clinicians that symptomatic hydrocephalus should be addressed before CIM. The experiences of treating hydrocephalic patients with concurrent CIM proved Chiari’s postulation to be seemingly valuable. Abundant research over the last few decades [3–7] has proven endoscopic third ventriculostomy (ETV) to be an effective alternative to shunting procedures in the treatment of obstructive hydrocephalus and special research concerning the application of ETV in hydrocephalic patients with concurrent CIM began in 1996 [8–13]. Unfortunately, the low CIM incidence hinders the improvement of such research; fewer than 60 cases have been reported worldwide (Table 1). There was not an obvious difference between the outcomes in adults and children in these studies. Some of these studies achieved complete alleviation of the symptoms induced by both hydrocephalus and CIM and even a reduction of tonsillar herniation and syringomyelia. The lack of consensus among previous studies left unanswered the question of how ETV addresses CIM and why it fails. Our retrospective study complemented previous work with the aim of identifying the efficacy of ETV through the outcomes, analysing the morphological variation and the mutual effect on both hydrocephalus and CIM, trying to interpret how ETV succeeds or fails. Additionally, this is the only report to date on a Chinese population.

**Table 1:** Review of the literature and current series

Author	Study time	No. of patients	Success rate	Follow-up time (month)
Fukuhara et al. [14]	2000	5	2/5	Range 3–62.8
Decq et al. [15]	2001	5	5/5	Mean 50.39 (range 14.27–109.37)
Hayhurst et al. [16]	2008	16	15/16	Mean 42 (range 6–96)
Massimi et al. [9]	2011	15	15/15	Mean 35 (range 15–86)
Woodworth et al. [17]	2011	10	Not reported	Mean 60 (range 24–108)
Salvador et al. [18]	2014	4	3/4	Mean 104.1 (range 18–164)
Current series	2015	10	8/10	Mean 92 (range 24–163)

## Materials and methods

### Clinical features

The study group was composed of ten inpatients consecutively treated for symptomatic hydrocephalus associated with CIM by ETV in our single neurosurgical institute from October 2002 until May 2012. Physical examinations and magnetic resonance imaging (MRI) (1.5 T) were carried out before surgery. The diagnosis of CIM was defined as a tonsillar herniation greater than 5 mm below the foramen magnum on sagittal MRI images. We classified the presented symptoms into “hydrocephalic symptoms” (for example, signs of increased intracranial pressure, cranial nerve VI palsy, developmental delay, gait disturbances, urinary incontinence, or macrocrania) and “classic CIM-related symptoms” (for example, suboccipital headache, ataxia, or disturbances of spinal cord function). None of the patients underwent the insertion of cerebrospinal fluid (CSF) diversion devices or any decompression to address the CIM before ETV. The mean age was 28.14 years (range 0.75–55 years) when ETV was performed.

### Treatment

All operations were performed by two experienced neurosurgeons via a rigid 0° endoscope (Karl Storz GmbH & Co., Tuttlingen, Germany) with an operating sheath (outer diameter 6.5 mm, working length 13 cm). Being shunt free and without hindbrain decompression at the last follow-up was defined as effective.

### Neuroimaging studies

The morphology of hydrocephalus and CIM was assessed in the image workstation, according to Evan’s Index (EI), the third ventricle transverse diameter (V3d), fourth ventricle sagittal diameter, and the extent of tonsillar herniation. Other findings were recorded concerning the expansion of the fourth ventricle, obstruction of CSF flow (by cine MRI), and the syrinx. Postoperative data were recorded at the last follow-up. Statistical analysis was performed using Student’s *t* test with significance set at *p* < 0.05.

## Results

### Preoperative findings

The clinical findings in these cases are summarized in Table 2. Nine patients complained of the main symptoms of hydrocephalus, including frontal headache, macrocrania, developmental delay, urinary incontinence, paroxysmal unconsciousness, nausea, and gait disturbance. Classic symptoms related to CIM or syringomyelia, including suboccipital headache and numbness of an upper limb, presented in only two patients. Aqueductal stenosis was evident in one patient, expansion of the fourth ventricle was evident in four patients, stenosis of Magendie’s foramen was observed in five patients, and occlusion of the cisterna magna was observed in one patient. No obstruction of the foramen magnum was identified.

**Table 2:** Clinical characteristics of ten hydrocephalic patients associated with Chiari I malformation

Case	Age (years)	Sex	Presented syndromes		Follow-up (months)	Outcome
			Hydrocephalus	CIM and syringomyelia		
1	42	Female	Frontal headache		72	Hindbrain decompression
2	27	Male	Headache, vomiting		157	Improved
3	1.67	Female	Macrocrania, developmental delay		111	Improved
4	0.75	Male	Macrocrania, urinary incontinence, paroxysmal unconsciousness		134	Improved
5	40	Female	Headache, nausea, gait disturbance	Numbness of upper limb	24	Deterioration
6	34	Female		Suboccipital headache	52	Improved
7	34	Male	Frontal headache		79	Improved
8	6	Male	Macrocrania, headache		86	Improved
9	41	Female	Paroxysmal frontal headache, nausea		163	Resolved
10	55	Female	Frontal headache		42	Stable
Mean	28.14				92.00	

### Postoperative complications

No surgical mortality or permanent complications occurred. A small intra-ventricle haematoma in one patient was observed via postoperative MR imaging after ETV, and the patient was discharged from the hospital without symptoms after conservative treatment. None of the patients died until the last follow-up.

### Long-term outcome

The mean follow-up period in this series of patients was 92 months (range 24–163 months). Among the ten patients, six experienced instant improvement of symptoms, one saw complete resolution of symptoms, and one maintained stable symptoms without progressive treatment due to economic and family reasons. The remaining two patients were identified as failures due to the deterioration of symptoms or the need for secondary surgery. Ultimately, the effective ratio of ETV was 80% (8/10). Interestingly, one patient with ventriculomegaly but asymptomatic hydrocephalus only complained of suboccipital headache, and ETV apparently relieved the symptoms. In contrast, another patient who only claimed to have a frontal headache subsequently underwent a hindbrain decompression procedure 7 years after the ETV.

### Neuroimaging findings

Pre- and postoperative MRI findings are listed in Table 3. The mean extent of tonsillar herniation were reduced from mean 9.6 to 9.0 mm after surgery but without significant differences (*p* = 0.054) (via Student’s *t* test). The transverse diameter of the third ventricle exhibited a reduction that was significant (*p* = 0.028). CSF flow was visualized on both sides of the orifice of the third ventriculostomy via cine MRI in all patients, confirming its patency and function.

**Table 3:** Neuroimaging findings of ten hydrocephalic patients associated with CIM

Case no.	Extent of tonsillar herniation (mm)		Extent of hydrocephalus						Other findings
			Evan’s Index		V3 transverse diameter (mm)		V4 sagittal diameter (mm)		
	Pre-op	Post-op	Pre-op	Post-op	Pre-op	Post-op	Pre-op	Post-op	
1	10.8	10.6	0.38	0.36	10.3	8.8	11.2	10.5	Normal V4 stenosis of MF, occlusion of the cisterna magna
2	13.7	13.5	0.35	0.35	11.3	9.4	14.6	14.7	Normal V4
3	7.6	7.4	0.58	0.54	9.9	8.9	8.8	9.3	Normal V4
4	7.2	7.1	0.52	0.59	11.2	13.3	8.2	8.1	Normal V4
5	6.5	6.6	0.49	0.50	19.2	19.0	12.4	16.5	Platybasia, expansion of V4, stenosis of MF
6	8.9	8.3	0.42	0.40	15.9	14.1	29.1	24.3	Expansion of V4, stenosis of MF
7	11.1	7.5	0.36	0.39	11.5	12.2	8.8	11.1	Normal V4, stenosis of aqueduct
8	8.3	8.1	0.44	0.42	16.4	13.9	14.3	13.9	Expansion of V4, stenosis of MF
9	9.5	9.0	0.38	0.32	12.2	6.8	22.0	12.8	Normal V4
10	12.2	11.7	0.40	0.40	16.5	13.8	11.8	11.5	Expansion of V4, stenosis of MF, cervical syrinx
Mean	9.6	9.0	0.43	0.43	13.4	12.0	14.1	13.3	Normal V4
*p* value	0.054		0.675		0.028		0.24		

*MF* Magendie’s foramen

## Discussion

When the significant clinical signs and neuroimaging findings of hydrocephalus are confirmed in patients with mild symptomatic or asymptomatic CIM, treatment should be initiated to address the hydrocephalus responsible for the increased intracranial pressure. This tendency is fairly common among neurosurgeons. ETV, which has already become a valuable alternative to shunting in treating hydrocephalus, was therefore introduced to manage the hydrocephalus associated with CIM.

Since the first descriptions of the treatment by Nishihara et al. in 1996 [12], we reviewed the subsequent literature (excluding case reports) that has reported on the management of hydrocephalus associated with CIM via ETV [9, 14–18] (Table 1). The success rate is not consistent and is in the range of 40–100%; in this study, it was 80%. The studies with large samples of mixed types of hydrocephalic patients seemed to result in higher failure rates than studies with small sample sizes. Fukuhara et al. reported that Chiari type I malformations was an important factor in failure rates, although without significance in a multivariate analysis [14]. In several specialized studies, ETV was effective in controlling hydrocephalus, as nearly all series reported a shunt-free rate of 100%, even though a subsequent hindbrain decompression may be required to resolve CIM [16]. Thus, the key factor determining the outcome of ETV remains to be explored.

Considering the seemingly low incidence of two diseases presenting simultaneously on admission, we cannot prove that hydrocephalus would inevitably cause CIM or vice versa. However, the mutual effect cannot be ignored. In fact, the first theory of CIM postulated by Chiari stated that CIM was attributed to the supratentorial pressure exerted by hydrocephalus. This effect exerted on CIM by hydrocephalus has been observed in several cases. In a study of the natural history of 22 paediatric patients with incidentally recognized CIM, the clinical signs of the malformation developed only in the two patients with associated mild hydrocephalus after a mean of 5.9 years’ follow-up [19]. The impact from CIM remained indeterminate. Majority of researchers attribute the exacerbation of hydrocephalus to obstruction of the fourth ventricle outlet and the impact of a narrow magnum foramen, which are both derived from the descending tonsillar. A narrow magnum foramen influences the CSF association between the cranial cavity and the spinal canal, which is an important condition to maintain the normal pressure and flow of CSF. Because the pulsatile blood flow in the vessel causes the pulsatile pressure of CSF, the dissociation of CSF circulation at the craniocervical junctional zone stops the pressure transmission, which then decreases the compliance of both the encephalon and spinal cord [20].

The results of our ETV research show that the procedure worked effectively in most patients (80%) with hydrocephalus and CIM, but there was a failure in a minority of the subjects. First, we must identify which part of the above correlation can be affected by ETV. ETV bypassed the third and fourth ventricles to release the pressure of the supratentorial cavity, which meant decreasing the above pressure due to hydrocephalus. Similarly, the obstruction of the fourth ventricle outlet also can be bypassed via ETV. This was observed in our study. After the resolution of hydrocephalus, there was a reduction of the tonsillar descent, as described in other reports [8, 9, 11, 16]. The average of the tonsillar descent reduced from 9.6 to 9.0 mm after our ETV; Hayhurst et al. [16] reported an average reduction of 15 to 13.3 mm, and Massimi et al. [9] reported an average reduction of 12.7 to 8.3 mm.

However, the impact of the narrow magnum foramen would not be alleviated by ETV, except that the reduced tonsillar herniation enlarges the cisterna magna, which results in the recovery of the CSF association. As expected, an overcrowded posterior fossa was found in nearly all CIM patients and remained an important aetiological factor. The concept of “cephalo-cranial disproportion”, or disproportionate intracranial volume and skull volume, was introduced by Hoffman and Tucker in 1976 to describe this overcrowding [21]. Naturally, the skull volume cannot be improved by ETV, but the intracranial volume may be. Nevertheless, Massimi et al. postulated a subtype of concurrent CIM and hydrocephalus with “reversible disproportion” [22]. These patients presented an expansion of the fourth ventricle with the normal posterior fossa, which caused the relative enlargement of the cerebellum. However, the disproportion can be reversed after operation if the fourth ventricle shrinks to normal size. From this perspective, if ETV addressed the hydrocephalus and shrank the fourth ventricle at the same time, the overcrowding of posterior fossa would be reversed as well.

According to a previous analysis, ETV is effective in the mitigation of hydrocephalus and part of CIM; more importantly, it blocks the vicious cycle. Thus, the decisive factor of the outcome should be whether ETV can resolve the major cause of the tonsillar herniation. Nevertheless, it is still difficult to identify the primary reason, whether it is supratentorial pressure from hydrocephalus or an overcrowded posterior fossa. Case 5 of our study did not experience the reduction in herniation after ETV. This means that the cephalo-cranial disproportion caused by posterior fossa hypoplasia, such as the platybasia of case 5, dominates the tonsillar herniation. Additionally, ETV is still limited in its ability to treat different types of hydrocephalus, such as communicating hydrocephalus and normal pressure hydrocephalus. Case 1 was the only patient who presented stenosis of Magendie’s foramen without expansion of the fourth ventricle. The cine MR of case 1 also presented the dissociation of CSF circulation due to stenosis of Magendie’s foramen and occlusion of the cisterna magna a year after ETV. We speculate that both explanations related to the tonsillar hernia were addressed by ETV. The occlusion of the cisterna magna was the reason for the failure, which prohibited the predicted recovery of CSF association resulting from ETV. Seven years later, a subsequent hindbrain decompression operation on case 1 finally overcame the limitation of ETV and resolved the overcrowding of the posterior fossa.

In addition, the assessment of “cephalo-cranial disproportion” was not easy to realize without a common and unified method. It is thus difficult to clarify the extent to which the overcrowded posterior fossa would dominate the herniation.

Endoscopic third ventriculostomy provides an effective treatment for hydrocephalus with concurrent CIM. This outcome requires addressing the major cause of the tonsillar herniation and the limitations of ETV in treating hydrocephalus. The effectiveness of ETV depends on proper criteria for patient selection.
